# No error on the side of safety: No representational momentum for auditory looming stimuli

**DOI:** 10.3758/s13423-026-02868-w

**Published:** 2026-02-17

**Authors:** Simon Merz, Vanessa Förster, Daniel Rupp, Tabea Wächtershäuser, Christian Frings, Charles Spence, Hauke S. Meyerhoff

**Affiliations:** 1 Department of Psychology, Cognitive Psychology, Trier University, Universitätsring 15, 54286 Trier, Germany; 2 Institute for Cognitive and Affective Neuroscience, Trier University, Trier, Germany; 3 Department of Psychology, Philipps-University Marburg, Marburg, Germany; 4 Department of Experimental Psychology, University of Oxford, Oxford, UK; 5 University of Erfurt, Erfurt, Germany

**Keywords:** Looming bias, Error management theory, Adaptive bias, Representational momentum, Motion perception, Speed prior account

## Abstract

The looming bias describes systematic differences in the perception of looming as compared to receding stimuli. To date, the most prominent and successful theory put forward to account for this bias is the adaptive bias theory, based on the more general error management theory framework, which argues for a perceptual bias for looming stimuli to err on the side of safety. We challenge this notion by providing evidence using the established probe comparison task from the representational momentum literature, in which the final stimulus configuration is probed. For intensity-changing sounds indicating looming/receding sound sources, no systematic overestimation in intensity change direction for the perceived final sound intensity of looming, approaching stimuli was observed. Across two auditory experiments using either classical sine wave (Experiment 1) or more complex tones (Experiment 2), we replicated the finding of no shift in intensity change direction for looming stimuli, even when accounting for general, change-independent biases. We provide an alternative framework, the speed prior account of motion perception, to explain the present, as well as further, currently unexplained findings in the literature.

## Introduction

We are constantly surrounded by dynamic, moving stimuli. The perception of these changing stimuli has been studied extensively, revealing various perceptual biases and experimental paradigms (e.g., the Fröhlich effect, Representational momentum, the Thompson effect, the flash lag/lead effect; see Hubbard, 2018b, for a review). A central argument is that looming stimuli – those that appear to be moving toward an observer – are particularly important, requiring action or response and thus receiving prioritized information processing. In auditory research, looming is often simulated using sounds that increase in intensity, mimicking an approaching sound source, while receding is simulated with sounds that decrease in intensity. This has revealed a robust perceptual asymmetry known as the ‘looming bias’ (Neuhoff, 2018): Looming sounds are perceived as changing more dramatically than equivalent receding sounds (Bach et al., 2008; Neuhoff, 1998; Neuhoff et al., 2009), and are anticipated to arrive sooner than would actually be the case (Neuhoff, 2001; Rosenblum et al., 1993; Schiff & Oldak, 1990). Theoretically, this is often explained by positing an adaptive bias for looming stimuli; this functional account proposes that natural selection may have favoured a perceptual system that overestimates the rate of change of approaching objects (see Neuhoff, 1998, 2001; see Neuhoff, 2018, for review and discussion). This systematic error, or bias, is thought to be adaptive because it creates a margin of safety for avoiding collisions, consistent with the broader framework of error management theory (Haselton & Buss, 2000, 2009). This bias is thought to be direction-specific (e.g., it is only observed for approaching motion) and beneficial for safe movement.

**Fig. 1:**
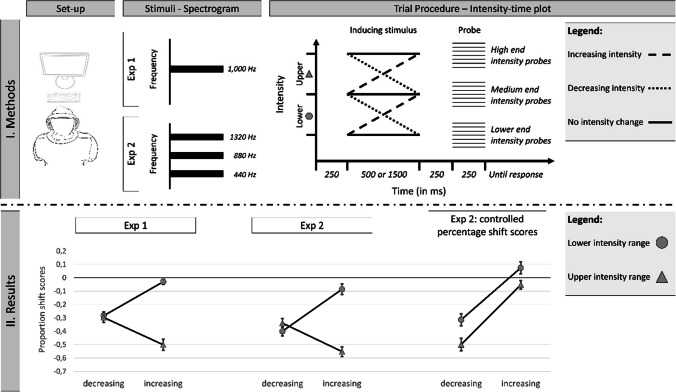
Visualization of the methods and results of the present study. Note: For the upper half (I. Methods), the experimental set-up, a spectrogram of the frequency composite of the two stimuli used in each of the experiments, and a trial procedure is depicted. Here, increasing intensity change stimuli are depicted with an elongated split line, decreasing intensity stimuli are depicted by a dotted line, and baseline, no change stimuli (only Experiment 2) are depicted with a continuous line. For the lower half (II. Results), mean proportion shift scores (with standard errors as error bars) as a function of intensity change direction (decreasing vs. increasing) and intensity range (lower vs. upper) are depicted for Experiments 1 and 2, as well as the controlled proportion shift scores calculated with baseline trials for Experiment 2 only

To date, tests of this adaptive bias, that is, a systematic shift in motion direction as a supportive mechanism to prevent delayed responses, have primarily been indirect (with Neuhoff, 2001, Experiments 2 A and B, being a notable exception discussed later). Typically, the bias is indirectly inferred from time-to-contact measurements (e.g., Schiff & Oldak, 1990), where an approaching object disappears and the observer indicates its expected arrival time, or from a higher perceived rate of intensity change for approaching versus receding stimuli (e.g., Ignatiadis et al., 2024; Neuhoff, 1998). However, the probe comparison task, common in research on Representational Momentum (e.g., Freyd & Finke, 1984), offers an established, two-alternative forced-choice method to directly measure the perceived final state of a changing stimulus (Hubbard, 2005, 2018a).^1^ In this task, a dynamic object is presented, followed by a probe stimulus with either the same or a slightly different final configuration (in or against the direction of change; see Fig. 1I). The proportion of ‘same’ responses is used to calculate shift scores, indicating the perceived stimulus configuration at offset (e.g., McCrink & Hubbard, 2017). This allows for a direct assessment of the perceived final stimulus configurations and can be used to test if the stimulus is perceived as shifted in the direction of change, as predicted by the adaptive bias framework (Neuhoff, 2018). The present study used established looming stimuli (intensity-increasing/decreasing sounds simulating approach/recession; Neuhoff, 1998; cf. Bach et al., 2008; for discussion, see Neuhoff, 2018) but probed perceived final intensity. While real-world auditory distance perception relies on multiple cues, including spectral information and the direct-to-reverberant energy ratio, dynamic intensity change is a primary and powerful cue for motion in depth (Kolarik et al., 2016) and the established manipulation for looming/receding stimuli (Bach et al., 2009; Neuhoff, 1998). While visual studies have explored the perceived final location of stimuli moving in depth (e.g., Hubbard, 1996; Nagai et al., 2002), a direct link to the auditory looming bias remains unclear, possibly due to these studies often showing a stronger localization bias for receding as compared to approaching stimuli, seemingly contradicting the idea of an adaptive advantage for looming stimuli (see Neuhoff, 2018, for discussion; see also the *General discussion* section).

### The present study

Across two experiments, we used the probe comparison task from the Representational Momentum literature (Hubbard, 2018a) in order to assess the perceived final intensity of an auditory target tone. Experiment 1 used standard sine wave stimuli (Neuhoff, 1998), while Experiment 2 used more complex tones (three frequencies; see Fig. 1I). Furthermore, as the perception of any dynamic event is likely dependent on its speed (e.g., Hubbard, 2005), we also included a manipulation of the rate of intensity change in our first experiment to explore whether this fundamental parameter modulates the perceptual bias. After the intensity-changing target and a short quiet interval of 250 ms,^2^ a probe stimulus was presented with either the same final intensity or a slightly more or less intense one (up to 6 dB). Participants judged if the final intensity matched the probe. This allowed us to calculate perceived final intensity and directly test the adaptive bias prediction of a forward shift (louder perceived final intensity for approaching stimuli). However, the results from both experiments contradicted this prediction, even after accounting for general judgement biases in Experiment 2. These findings directly challenge the adaptive bias account (Neuhoff, 1998), and instead align with the speed prior account (Merz et al., 2022), a recent theoretical development for spatially changing stimuli (see the *General discussion*).

## Experiment 1

### Methods

#### Participants

Because this was the first study to investigate a possible representational momentum effect for auditory stimuli with changes in sound pressure level/intensity, it was difficult to predict whether or not an effect would be found. As the reported biases (representational momentum, the reverse effect of offset repulsion, as well as looming bias) typically elicit medium to large effect sizes (d_z_ around 0.6), we aimed for at least 26 participants (α <.05; 1-β >.90; power analyses were run with G-Power 3.1.9.2, option ‘means: difference from constant’; Faul et al., 2009). To account for possible dropouts, 30 participants were tested. The final sample consisted of 30 students (26 female; one left-handed; age 18–26 years, mean age: 21.13 years) from the University of Trier who took part in this study in return for course credit. All of the participants reported normal hearing, gave written informed consent prior to participation, and the experimental procedures were conducted in line with the suggestions outlined in the Declaration of Helsinki and its later amendments.

#### Design

The participants were tested in a 2 × 2 × 2 × 7 design with the within-participants factors, intensity change (decreasing vs. increasing), intensity range (lower vs. upper), stimulus speed (slow: 1,500 ms vs. fast: 500 ms) and probe intensity (−6 vs. −4 vs. −2 vs. ± 0 vs. + 2 vs. + 4 vs. + 6 dB). Hereby, the factor probe intensity was used to compute the proportion shift scores (for details, see the *Data preparation* section), which were used as the dependent variable (for identical approaches, e.g., see Hubbard & Ruppel, 2014).

#### Apparatus and stimuli

Each participant was tested in isolation, either alone or with another participant in the same standard laboratory, but on different, opposite direction-facing workstations. The stimuli were generated with Audacity (version 2.3.0) and presented on a PC. All instructions and stimuli were presented via E-Prime (version 2.0). The auditory stimuli consisted of pure sine wave tones (1,000 Hz) presented using closed standard headphones (Creative Labs Fatal1ty HS-800 Gaming headset). The intensity of the presented stimulus changed continuously by 15 dB, changing over 500 ms or 1,500 ms (depending on the experimental condition).^3^ All sound intensities were measured on one side of the headphones using a XL2 Audio and Acoustic Analyzer with M4260 microphone (NTi Audio; Schaan, Liechtenstein).

#### Procedure

During each trial, an inducing stimulus and a probe stimulus were presented, and participants were asked to estimate whether the final intensity of the inducing stimulus was the same or different to the intensity of the probe stimulus (see Fig. 1I). Each trial was as follows: The trial started with the presentation of a cross from the center of the screen for 600 ms (to indicate the start of a trial), immediately followed by the presentation of the inducing stimulus. The inducing stimulus was presented either for 500 ms (fast condition) or for 1,500 ms (slow condition). After an interstimulus-interval (ISI) of 250 ms, the probe stimulus was presented for 250 ms. The ISI of 250 ms was chosen as this is the interval which was shown to exhibit the strongest forward shifts in vision (Freyd & Johnson, 1987; de Sá Teixeira et al., 2013) as well as audition (Freyd et al., 1990). The participants then identified via keypress (keys D and J) without any time restrictions whether the final intensity of the inducing stimulus was identical to, or different from, the intensity of the probe stimulus.

Two different intensity ranges were used. The intensity of the inducing stimulus was programmed to either change between 50 and 65 dB (lower intensity range; following final test with the actual set-up, the emitted intensities were 49–64 dB, respectively) or between 65 and 80 dB (upper intensity range; actual emitted intensities were 64–79 dB, respectively). Additionally, the direction of the intensity change was manipulated, that is, the intensity either increased (from 49 to 64 dB in the lower range, and from 64 to 79 dB in the upper range) or else decreased (respectively from 79 to 64 dB in the upper range or from 64 to 49 dB in the lower range). The intensity of the probe stimulus depended on the final intensity of the inducing stimulus, and was either identical (± 0 dB), shifted in intensity change direction (+ 2 dB, + 4 dB, + 6 dB), or shifted against intensity change direction (˗2 dB, ˗4 dB, ˗6 dB). For example, for the decreasing lower intensity condition, in which the final intensity of the inducing stimulus was 49 dB, the intensity of the probe stimulus was one of seven intensities (43 dB, 45 dB, 47 dB, 49 dB, 51 dB, 53 dB or 55 dB).

The experiment started with 12 practice trials, which were identical to the experimental trials and selected at random from all possible trial types. Overall, participants worked through 448 experimental trials (eight repetitions per condition). After every 40 trials, the participants were given the chance to take a break. In total, the experiment lasted for about 30 min.

#### Data preparation and analysis

To calculate the proportion shift scores, the three matching probe positions (i.e., ± 2, ± 4, and ± 6 dB) were related.^4^ For this, the proportion of same responses for the backward shifted probes (against change direction; probe with negative sign) was subtracted from the proportion of same responses for the forward shifted probes (in change direction; probe with positive sign). Then, these scores were averaged across the three different probe positions (6, 4, and 2). This represents the average difference of the proportion of same response between the forward shifted and backward shifted probes. To interpret the magnitude of these scores, a value of + 1 would indicate that participants always responded ‘same’ to probes shifted in the direction of motion and never to probes shifted against it, while a value of ˗1 would indicate the reverse. A value of ˗0.50 would thus represent a substantial shift against the direction of motion, for example indicating that participants were far more likely to judge a quieter probe as matching the final intensity than a louder one for an increasing intensity stimulus. These proportion shift scores were calculated for each participant and for each combination of the three factors (stimulus speed; intensity change; intensity range) separately. All of the experimental stimuli, files, and analyses scripts are openly available via the Open Science Framework (OSF) at: https://osf.io/k7w4p/?view_only=66a77a3b5492411d8ab9a601553d1643. See Table 1 for the proportion shift scores of all condition combinations. In the associated OSF project, a supplementary document with visualization of the raw proportion of same response for each experimental condition is provided.

**Table 1:** Mean proportion shift scores (standard deviations in brackets) as a function of experiment (1 vs. 2), intensity change (increasing vs. decreasing), stimulus speed (slow vs. fast), and intensity range (lower vs. upper) as well as baseline (48, 63 or 78 dB)

Experiment	Speed	Condition	Slow	
			Lower	Upper
1	Slow	Increasing	−0.01 (0.16)	−0.47 (0.29)
		Decreasing	−0.28 (0.21)	−0.33 (0.20)
	Fast	Increasing	−0.05 (0.12)	−0.54 (0.21)
		Decreasing	−0.29 (0.17)	−0.26 (0.25)
2		Increasing	−0.09 (0.22)	−0.55 (0.20)
		Baseline	−0.16 (0.18)	−0.50 (0.23)
		*Controlled proportion shift*^a^	*0.07 (0.26)*	*−0.05 (0.18)*
		Decreasing	−0.40 (0.19)	−0.34 (0.19)
		Baseline	0.09 (0.15)	−0.16 (0.18)
		*Controlled proportion shift*^a^	*−0.31 (0.26)*	*−0.50 (0.27)*

Proportion shift scores are presented, with standard deviations in brackets. Positive proportion shift scores indicate a judgement in intensity change direction (forward shift), negative proportion shift scores indicate a judgement against the direction of intensity change (backward shift). For the baseline condition, a positive score indicates a perceived higher intensity as presented, while a negative score indicates a perceived lower intensity then presented

^a^ The controlled proportion shift scores can be interpreted as the basic proportion shift scores (positive = judgement in intensity change direction; negative = judgement against intensity change direction) and is the difference between the proportion shift scores and the respective baseline condition

### Results and discussion

#### Intensity judgement

As can be seen in Table 1, a negative shift was observed in all conditions, in contrast to the prediction of the adaptive bias account, which would never predict a backward shift, but always a forward shift. A 2 (intensity change: decreasing vs. increasing) × 2 (intensity range: lower vs. upper) × 2 (stimulus speed: fast vs. slow) analysis of variance (ANOVA; see Table 1 for the means) was conducted. A significant main effect of intensity range was observed, *F*(1, 29) = 84.85, *p* <.001, ɳ_p_2 =.75, indicating a smaller backward shift in the lower condition (˗0.16) than in the upper condition (˗.40). Besides the shifts being negative, not positive, the direction of these effects (more negative effects in the upper condition) is contrary to the predictions of the adaptive bias account. Furthermore, no effect of intensity change (increasing: ˗0.27; decreasing: ˗0.29) was observed, *F*(1, 29) = 0.91, *p* =.347, ɳ_p_2 =.03. Please note as the adaptive biases specifically argue for a perceptual bias solely for increasing, looming stimuli, a difference would likely have been expected here from an adaptive bias perspective. The main effect of stimulus speed (fast: ˗0.29; slow: ˗0.27) was also not significant, *F*(1, 29) =.31, *p* =.585, ɳ_p_2 =.01.

The interaction between intensity change and intensity range was significant, *F*(1, 29) = 55.27, *p* <.001, ɳ_p_2 =.66 (for a visualization, see Fig. 1II). The observed main effect of intensity range was driven by the increasing, looming stimuli, as here a strong difference between the upper (˗0.50) and lower (˗0.03) values was observed, *t*(29) = 12.14, *p* <.001, *d* = 2.22, while no difference (upper: ˗0.30; lower: ˗0.28) was observed for decreasing, receding stimuli, *t*(29) = 0.30, *p* = 0.769, *d* = 0.05. Interestingly, once again, as looming stimuli in the upper range are likely perceived to be closer, a tendency for an overestimation would have been expected for these stimuli; however, the reverse pattern (even stronger underestimation) was observed. Additionally, the interaction between intensity change and stimulus speed, *F*(1, 29) = 5.29, *p* =.029, ɳ_p_2 =.15, indicated a tendency toward more negative shifts for faster speeds of approaching, increasing stimuli (slow: ˗0.31; fast: ˗0.27), *t*(29) = ˗1.84, *p* =.076, *d* = 0.18, while no difference was observed for the receding condition (slow: ˗0.24; fast: ˗0.29), *t*(29) = 0.96, *p* =.34, *d* = 0.19. None of the other interactions was significant, *F*s < 2.37, *p*s >.135. Taken together, the results of Experiment 1 are in direct contradiction to the predictions derived from the adaptive bias account (Neuhoff, 2018).

To ensure the robustness of the first experiment, a second study was conducted to try and replicate the findings with slightly different stimuli. Furthermore, to really assess the importance of the dynamic nature of the stimuli, baseline measurements of stimuli without intensity change were included. This addition addressed the potential influence of general, change-independent auditory localization biases (Getzmann & Lewald, 2007; Schmiedchen et al., 2013), a factor that complicates the interpretation of Neuhoff’s (2001) study, which used a direct measure of looming bias but lacked such baselines. Although Neuhoff’s data hinted at general biases (e.g., all dynamic, receding as well as looming stimuli were judged around a 5-m distance despite being presented at 20 m), the absence of baseline measures prevented a clear determination of the specific perceptual effects of the dynamic nature of the approaching and receding sound sources. Our inclusion of baseline measures in the second experiment aimed to overcome this limitation to purely focus on the influence of motion of the stimulus.^5^ To accommodate this new condition while at the same time maintaining a reasonable duration for the experiment, no manipulation of stimulus speed was included in Experiment 2.

## Experiment 2

### Method

#### Participants

Sample size considerations were as for Experiment 1, and the final sample (25 female; three left-handed; age 19–42 years, mean age: 22.28 years) consisted of 32 students from the University of Trier who took part in this study in return for course credit.^6^ As in Experiment 1, all of the participants reported normal hearing and gave written informed consent prior to their participation.

#### Apparatus and stimuli, procedure, design and analysis

The apparatus and stimuli, procedure, design and analysis were identical to Experiment 1 with the following exceptions. Complex tones were used instead of the sine wave tones used in Experiment 1. They were created by the superposition of harmonic oscillations whose frequencies are in an integer relationship to each other, in this case a composite of 440 Hz (see Fig. 1I, all sound files used in the two experiments are uploaded on the study’s OSF page). That is, the three frequencies were 440 Hz, 880 Hz and 1,320 Hz, all with the same amplitude. Furthermore, a ramp-up and ramp-down for the first/last 20 ms of every tone was included to avoid any clicking at the beginning or end of the sounds. The inducing stimulus was always presented for 1,500 ms. With the new tones, the final intensities differed slightly from Experiment 1. The lower intensity range was between 48 and 63 dB, the upper intensity range was from 63 to 78 dB. In contrast to Experiment 1, a baseline measurement in which the inducing stimulus did not change in intensity was added. For the three final intensities (48, 63 and 78 dB), an inducing stimulus which did not change in intensity was presented, in order to get a control estimate without any intensity change (Fig. 1I – trial procedure – continuous line). For those control trials, the same seven probe intensities were presented, and proportion shift scores were calculated in such a way that positive values indicated a perceived higher intensity, while negative values indicated a perceived lower intensity. Participants completed a total of 404 trials.

### Results and discussion

#### Intensity judgement

As in Experiment 1, all shift scores for the changing intensity stimuli were negative (see Table 1 for the means), once again in contrast to the predictions of the adaptive bias account. A 2 (intensity change: decreasing vs. increasing) × 2 (intensity range: lower vs. upper) ANOVA was conducted, comparable to Experiment 1 with the exception that the speed factor was dropped. Importantly, the results closely resembled those of Experiment 1 descriptively as well as statistically (see Fig. 1II), with the non-significant main effect of intensity change, *F*(1, 31) = 2.27, *p* =.142, ɳ_p_2 =.07, and the two significant effects of intensity range* F*(1, 31) = 38.08, *p* <.001, ɳ_p_2 =.551*,* as well as the interaction, *F*(1, 31) = 74.07, *p* <.001, ɳ_p_2 =.71, indicating similar influences to those in Experiment 1. In order to directly test if both experiments actually indicate similar results, a 2 (intensity change: decreasing vs. increasing) × 2 (intensity range: lower vs. upper) × 2 (Experiment: 1 vs. 2) ANOVA was conducted (see Table 1 for the means). For Experiment 1, only the comparable slow condition was used. Importantly, the factor Experiment did not interact with any of the effects, *Fs* < 1.49, *p* >.228, and a significant main effect of Experiment was observed, *F*(1, 60) = 4.51, *p* =.038, ɳ_p_2 =.070, with even more negative shift scores for Experiment 2 (˗0.35) than for Experiment 1 (˗0.28). Once again, this result is against the predictions of the adaptive bias account which would expect stronger/more positive scores for complex tones (Experiment 2) than simpler pure sine wave tones (Experiment 1).

#### General biases analysis

In a first step, baseline trials were separately analyzed, to see whether general, change-independent biases exist. Therefore, proportion shift scores for the three final intensities (48 dB, 63 dB and 78 dB; see Table 1 for descriptive values – please note that the decreasing upper and increasing lower condition combination had the same final intensity – 63 dB – and subsequently the same baseline scores) were analyzed in a one-factorial ANOVA with the factor baseline (48 dB, 63 dB or 78 dB). Note that positive values indicate a perceived louder intensity, and negative values indicate a perceived quieter intensity. The main effect of baseline was significant, *F*(2, 62) = 77.59, *p* <.001, ɳ_p_^2^ =.72, with the lowest intensity being perceived as louder (48 dB: 0.09), *t*(31) = ˗3.18, *p* =.003, *d* = 0.56, whereas middle intensity (63 dB: ˗0.16), *t*(31) = 4.89, *p* <.001, *d* = 0.87, and upper intensity stimuli (78 dB: ˗0.50), *t*(31) = 12.40, *p* <.001, *d* = 2.19, were perceived as quieter than presented. This indicates general, change-independent perceptual biases (in line with Getzmann & Lewald, 2007, and Soballa et al., 2025), similar to centering/contraction biases reported across the different sensory modalities (Merz et al., 2020; Schmiedchen et al., 2013; Soballa et al., 2025; Steenbergen et al., 2014), which might have its physiological basis in dynamic range adaptation (e.g., Dean et al., 2005; Wen et al., 2009). Therefore, the proportion shift scores from the increasing/decreasing tone trials were adjusted by the general proportion shift scores observed for the respective end intensity (baseline condition in Table 1), and these controlled proportion shift scores were then used as the new dependent variable.

A 2 (intensity change: decreasing vs. increasing) × 2 (intensity range: lower vs. upper) ANOVA with controlled proportion shift scores as the dependent variable was conducted (for an illustration, see Fig. 1). Once again, the main effect of intensity change was significant, *F*(1, 31) = 60.48, *p* <.001, ɳ_p_^2^ =.66, with decreasing stimuli leading to a backward shift (˗0.41) and increasing stimuli being judged accurately (0.01). While a general difference between increasing/decreasing intensity is in line with the adaptive bias idea, the observation of no shift in change direction is not. The main effect of intensity range was significant, *F*(1, 31) = 16.89, *p* <.016, ɳ_p_^2^ =.35, with lower intensity stimuli leading to a backward shift (˗0.12) and higher intensity leading to an even stronger backward shift (˗0.28), once again against the prediction of the adaptive bias account. The interaction between intensity change and intensity range disappeared when controlled shift scores were used, *F*(1, 31) = 0.869, *p* =.360, ɳ_p_^2^ =.03.

## General discussion

Across two experiments, our data do not align with the idea of an adaptive, perceptual bias for approaching stimuli, as is typically argued in the looming bias literature (e.g., Neuhoff, 1998, 2001, 2018). In the present study, we used an established measure of the perceived end state of dynamically changing stimuli, the probe comparison task frequently used in the representational momentum literature (cf. Hubbard, 2005, 2018a), to directly evaluate the adaptive bias account for approaching stimuli in audition. The results deviate from the predictions of the adaptive bias account as even though differences for the different motion direction were observed, most prominently, no shift in change direction was observed. In certain conditions, specifically for receding sounds, the highest proportion of ‘same’ responses occurred at our most extreme negative probe (˗6 dB), suggesting a potential ceiling effect where the actual perceived final intensity may have been shifted even further against the direction of change (for details, see the Online Supplementary Material in the project’s OSF page). However, this limitation does not challenge our primary conclusion, as it further reinforces the absence of a forward representational momentum effect for looming stimuli. Furthermore, higher intensity sounds elicited weaker/more negative shifts, against the predictions and data from the adaptive bias account (Neuhoff, 1998).

Beyond the findings reported here, the ‘decruitment’ literature (Canévet & Scharf, 1990; Canévet et al., 1999; but see Neuhoff, 1999) offers further evidence that the adaptive bias account may not fully explain perception. Specifically, in decruitment studies, where participants match perceived intensity to a numerical value, the perceived difference between the onset and offset of decreasing intensity tones is typically larger than for increasing tones. This contrasts with the looming bias literature, which generally finds a greater overall perceived change for increasing intensity sounds as compared to decreasing ones (Olsen, 2014). While some researchers suggest that the different tasks (intensity matching in decruitment vs. overall change estimation in looming) engage distinct processing mechanisms (Canévet et al., 1999; but see Neuhoff, 1999), we propose a critical difference lies in the stimulus presentation time: seconds in looming studies versus tens of seconds or minutes in decruitment studies. We offer an alternative theoretical explanation grounded in the speed prior account (Merz et al., 2022). Our argument is that the looming literature typically employs faster stimulus speeds (e.g., 15- to 30-dB changes in hundreds of milliseconds to a few seconds, e.g., > 5 dB/s; Bach et al., 2008; Ignatiadis et al., 2024; Neuhoff, 1998), whereas the considerably slower speeds used in decruitment studies (e.g., 15- or 30-dB changes in tens of seconds to a few minutes, e.g., < 1 dB/s).

The speed prior account posits that expectations about stimulus speed influence perception (for general arguments about the importance of expectations for perception, see Knill & Pouget, 2004; Knill & Whitman, 1996; Ma et al., 2023). When actual speed differs from expected speed, perceptual distortions arise: faster speeds lead to length contraction, while slower speeds lead to length extension (Merz et al., 2022), which might align with typically faster speed in the looming literature, and slower speeds within the decruitment literature. Furthermore, we propose, but have not yet explicitly tested, that the relative weighting of the speed expectation (the prior) and the incoming sensory input differs for approaching and receding stimuli. This proposal is grounded in the extensive literature demonstrating that approaching stimuli are more salient and preferentially capture attention compared to receding stimuli (Franconeri & Simons, 2003; Gray, 2011; Ho et al., 2013). This prioritized processing can be conceptualized within a Bayesian framework as a higher weighting of the sensory likelihood for more salient or potentially threatening stimuli, leading to a perception that is more faithful to the sensory input and less biased by the internal prior. In contrast, less salient receding stimuli would be more susceptible to the influence of the speed prior, resulting in stronger perceptual distortions. Recent neuropsychological evidence indicating top-down projections that bias the processing of looming sounds may provide the neural correlate for this proposed differential weighting (Ignatiadis et al., 2021).

The speed prior account also offers an alternative explanation for findings from visual depth localization studies (Hubbard, 1996; Nagai et al., 2002), which have shown mixed results like forward shifts (representational momentum) and backward shifts (offset repulsion). Unlike Hubbard’s (1996) composite explanation involving boundary extension, which our data do not consistently support given the observation of a contraction bias for stationary, non-changing sounds, the speed prior account aligns with the observed tendency for a stronger localization bias for receding stimuli in those studies. This is because the speed prior account posits a greater influence of prior expectations for receding stimuli, leading to stronger localization biases, thus offering a compelling alternative to previous interpretations.

The speed prior account offers a unifying account for findings across the looming and decruitment literatures, as well as our present study. For fast stimuli, it predicts a stronger perceived change for looming stimuli (consistent with the looming bias; Neuhoff, 1998, 2018), while for slow stimuli, it predicts a weaker perceived change (consistent with decruitment; Canévet & Scharf, 1990; Canévet et al., 1999; for review, see Olsen, 2014). Our observation of general negative shifts, particularly with our fast stimuli (15 dB in 500/1,500 ms, similar to looming studies), supports this notion. Furthermore, the stronger negative shifts for receding stimuli and the trend for faster approaching stimuli to have more negative shifts align with the account’s predictions.

While further research with extensive speed manipulations and contextual adaptations of speed expectations is needed (Merz et al., 2022, 2023, 2024), our results provide a promising initial validation. In conclusion, our findings challenge the adaptive bias explanation for the looming literature (Neuhoff, 2018) and are in line with the speed prior account as an alternative framework for understanding motion in depth perception, potentially resolving discrepancies between the decruitment and looming literatures.

## Data Availability

Available in the Open Science Framework (OSF) repository: https://osf.io/k7w4p/overview?view_only=eacffa88e7a14b52a12409d714113c70

## Footnotes

1 Time-to-contact estimates correlate with perceived final location judgements for horizontally moving objects approaching a barrier (Gray & Thornton, 2001). Furthermore, the probe comparison task has successfully been used to measure perceived final pitch of auditory stimuli (e.g., Freyd et al., 1990; Kelly & Freyd, 1987).

2 Please note that previous research in vision (Freyd & Johnson, 1987; de Sà Teixeira et al., (2013) and audition (Freyd et al., 1990) has shown that an interstimulus interval between inducing stimulus and probe of 200–300 ms typically elicits the strongest forward bias, therefore increasing the chance of observing a forward shift if it existed. Additionally, a complete reversal of an existing effect depending on the RSI was not found consistently, indicating that RSI might influence the size of the effect, but does not qualitatively change the interpretation of the results. Therefore, one fixed retention interval of 250 ms was chosen.

3 Please note that sensory adaptation to increasing/decreasing intensity is rather unlikely to influence the observed results of our study given the rather short presentation times used (500/1,500 ms) compared to adaptation periods of 2 s in studies of sensory adaptation (e.g., Reinhardt-Rutland & Anstis, 1982).

4 Those readers who are more familiar with the probe comparison task/representational momentum literature are likely wondering why the more familiar weighted mean averages were not calculated in the present study. The reason is that human auditory perception is better represented on a logarithmic scale (the dB scale), not on a linear one (as, e.g., in the case of visual spatial perception, where distance is measured linearly, e.g., the metric system). For example, a 6- to 10-dB increase or decrease is typically perceived as a doubling/halving of intensity, indicating the logarithmic relation of sound pressure change and human perception (for an extensive discussion, see Moore, 2012). That is, a 6-dB increase in intensity corresponds to a doubling of sound pressure level (measured in Pascals – a linear scale), while a 6-dB decrease corresponds to 50% of sound pressure level. Therefore, as human perception relates to the logarithmic dB scale, the experiment was designed in dB, with the most extreme probes being ± 6 dB, as this related to a substantial intensity increase which is well perceived, and the least extreme probe being ± 2 dB, as this is still more than the smallest just noticeable intensity change discussed in the literature (see Moore, 2012, for discussion). Given the logarithmic relation between sound pressure and human hearing, we refrained from using the classical weighted mean scores as for these a linear relation between the probe locations would be needed, and therefore use the perceived proportion shift which do not assume a linear relation between probe positions (see McCrink & Hubbard, 2017). That is, for this analysis, only the equally spaced probe positions (e.g., the 6 dB louder and quieter) are related, however, no equal spacing between the probes is assumed (as would be with weighted average scores).

5 Please note that given that the adaptive bias account emphasizes the direct perception of looming stimuli in everyday contexts, Neuhoff’s (2001) decision to omit baseline measurements might stem from the understanding that adaptive behavior is driven by the immediate perception of motion, not by comparisons to non-dynamic stimuli. From an evolutionary perspective, underestimation of an approaching threat carries a significant cost, whereas relative overestimation in comparison to a static object is less critical.

6 Due to an organizational error, two more data sets were collected than pre-planned.

## Code availability

Available in the OSF repository: https://osf.io/k7w4p/overview?view_only=eacffa88e7a14b52a12409d714113c70
